# ‘Obviously, you can’t outright ask’: what are the barriers and facilitators to discussion of spiritual health within social prescribing? A study using semi-structured interviews

**DOI:** 10.1186/s12875-025-03060-0

**Published:** 2025-12-10

**Authors:** Ishbel Orla Whitehead, Mark Adley, Alexandra Thompson, Philip Mordue, Amy O’Donnell, Barbara Hanratty

**Affiliations:** Population Health Sciences Institute, Faculty of Medical Sciences, Newcastle University, Newcastle upon Tyne, UK

**Keywords:** Spiritual health, Social prescribing, Spirituality, Primary care

## Abstract

**Background:**

Social prescribing aims to provide holistic care to patients and meet needs that expand beyond the biomedical model. Holistic care includes spiritual health. However, the understanding of social prescribers’ attitudes towards discussing spiritual health with their patients is limited. This study aimed to understand how spiritual health currently fits into social prescribing and explore barriers and facilitators to incorporating spiritual health within social prescribing practice.

**Method:**

Twelve social prescribers were interviewed online, using semi-structured interviews. These interviews were an hour long and covered aspects of spiritual health within social prescribing, as well as barriers and facilitators to the discussion of spiritual health within social prescribing. Thematic analysis was used to analyse the interviews by three researchers.

**Results:**

Currently, social prescribers use community faith-based organisations mainly for non-spiritual holistic support, especially around practical items. They identified an overlap and limited differentiation between spiritual health and mental health. When a patient discloses spiritual health needs, social prescribers felt they were open to helping patients access help.

Barriers and facilitators to the discussion of spiritual health included: viewing religion as a sensitive topic and a subsequent fear of ‘deep conversations’; concern about harm or offence to vulnerable patients; social prescribers’ comfort and confidence with the topic being part of their role, as well as discordance/concordance with patient beliefs.

**Conclusions:**

Social prescribers appeared very open to the topic of spiritual health; however many felt more confident with non-traditional-Western spiritual activities such as yoga or meditation, and using community faith-based organisations for non-spiritual support. Bespoke training for those in primary care could address barriers to the inclusion of spiritual health in primary care, but a systemic cultural approach is needed.

**Supplementary Information:**

The online version contains supplementary material available at 10.1186/s12875-025-03060-0.

What is ‘spiritual health’? Spiritual health is a broad concept, as diverse as people themselves. In this work, spiritual health was not defined and left to the participant to define themselves. The authors use a definition of spiritual health derived from UK General Practitioners and further developed with social prescribers: self-actualisation, peace, purpose and meaning; transcendence, connectivity and relationships beyond the self; and expressions of spirituality. [Whitehead O, Jagger C, Hanratty B What do doctors understand by spiritual health? A survey of UK general practitioners BMJ Open 2021;11:e045110. 10.1136/bmjopen-2020-045110].

## Introduction

Within UK primary care, social prescribing has been developed to address health inequalities and encourage personal engagement with health through improved support to access non-clinical, community-based activities [1, 2]. The social prescribing model uses embedded local link workers who serve as a referral point for General Practitioners (GPs, or family doctors) and other primary care professionals to act as a ‘bridge’ connecting patients to community, non-medical, socially-oriented interventions with appropriate resources to address their health and care needs. Link workers possess comprehensive knowledge of available services from community and third sector organisations, known as ‘asset’ and ‘place’ based interventions [2, 2–4]. Meaning, purpose, and connections are components of spiritual health [5], which itself constitutes a significant aspect of health and wellbeing [6]. 

Existing evidence suggests that spirituality and religiosity can positively impact patients’ health and social care outcomes, including enhanced longevity [7, 8], improved outcomes for those with mental illness [9, 10], dementia [11], and a potential reduction in stigma and loneliness for those with HIV [9]. Rehabilitation after cardiac illness may be enhanced by engagement with religious services [9]. In contrast, neglecting spiritual needs can be detrimental to health [12], including higher levels of pain [13, 14], greater mortality [15], wishes for a hastened death [16], and reduced wellbeing [17]. Salutogenesis is a core concept of social prescribing referring to the ‘creation of health’ [18, 19]. Salutogenesis is defined as including the spiritual aspects of health [20]. Alongside this is one of the underlying principles of social prescribing – to find out from patients *what matters to you?* [1] with this question framed in contrast to the biomedical model which defines patients by their illness (or what’s the matter *with* you?). However, despite awareness of the importance of spiritual health within salutogenesis, spiritual care providers (such as, but not limited to, chaplains, shamans, spiritual healers, parish nurses etc.) or faith communities appear to be missing from current social prescribing initiatives [3, 19, 21]. 

Our recent survey of 171 UK social prescribers found that although social prescribers feel comfortable with the topic, barriers to the inclusion of spiritual health in social prescribing were: a perceived need for patient cues and training; fear of causing offence or upset; and pre-existing practitioner attitudes towards and/or interest in the topic Whitehead et al., 2025, submitted. In that survey, social prescribers identified a need for further training in spiritual health, and further exploration is needed to explore what would be useful for overcoming barriers. This study aims to investigate the barriers and facilitators to the inclusion of spiritual health within UK social prescribing, via semi-structured interviews.

### Aim

To understand the barriers and facilitators to the inclusion of spiritual health within social prescribing.

### Objectives

- To deepen our understanding of the current inclusion of spiritual health in the social prescribing offer.
- To understand perceptions of barriers and facilitators to inclusion of spiritual health in the social prescribing offer.
- To explore training needs of people working in social prescribing around discussion of spiritual health.

## Method

### Design and setting

This study was grounded within a pragmatic research paradigm, which allows investigation of topics such as the experience and knowledge of discussing spiritual health, and applications of that knowledge. A qualitative study design was selected, that aimed to explore participants’ understanding of spiritual health within the context of their role and their related training needs. Data were collected via semi-structured interviews conducted between December 2024 and February of 2025 using Microsoft Teams [22] or Zoom [23] video conferencing software A risk assessment was undertaken to ensure participant and researcher safety (Supplementary material 1, pages 7–8). Steps were taken to identify and exclude bogus participants (Supplementary material 2).

### Recruitment and sampling

Twelve participants aged above 18 years were recruited purposefully from different ethnic groups and geographic areas to give a range of potential views and experience. Participants were purposefully recruited who had an interest in the topic, in order to generate data. Recruitment took place via email. Invitations were sent out throughout the UK to care boards, practices, health boards, clinical research networks, and shared via professional networks and social media. An opt-in approach was used in which potential participants had to indicate a positive interest in being contacted about the research, with a £20 voucher offered in gratitude. Eligibility criteria are detailed in Table 1 below.

**Table 1:** .

**Inclusion criteria**	**Exclusion criteria**
• Resident in the UK • Work in primary care • See their role as within ‘social prescribing’ • Have direct patient contact • Have an interest in the topic of spiritual health	• Those who have no direct patient contact • Those who see their role as purely administrative (for example receptionists trained in ‘care navigation’) • No exclusion based on sex, age, ethnicity, disability, maternity, etc.

### Data collection techniques

Data were collected in semi-structured interviews conducted by IOW and AT using a topic guide. (Supplementary Material 3) Consent for interview, data collection and publication were obtained from participants. Interviews used open-ended questions and lasted an average of 50 min (range 36–69 min). The interview guide was discussed with the SHARP (spiritual health awareness and recommendations in primary care) project advisory group and piloted with three social prescribers associated with the team. Introductory and open questions were followed by structured questioning to collect professionals’ views on how spiritual health was included within their roles. Interviews were audio-recorded within Zoom or Teams, with recordings saved onto a secure server before being transcribed, either by a professional transcription service or using software-based transcription. Identifiable data were anonymised or removed after transcription.

### Data analysis

IOW, MA, and AT analysed interviews between 6th January and 3rd February 2025. A reflexive thematic analysis was conducted using hand-coding techniques, as in Braun and Clark’s guide [24, 25]. This involved immersion and familiarisation with the data, coding, development of themes, and report writing [24], using printed hard copies of anonymised transcripts, colour coding, scissors and pens. The three researchers met on 3rd January to discuss and refine the analysis. Researcher positionality was acknowledged and reflected upon [24]. IOW and BH work within primary care as GPs; MA has worked as a social prescriber for over five years; AT is a psychologist; PM trained as a radiographer and AOD is an experienced researcher. The team convened regularly to share their diverse experiences as healthcare practitioners from various multidisciplinary team and academic perspectives, united by their interest in holistic health, to consider different viewpoints on the data. Software programmes MAXQDA [26] and NVivo [25] were used for clustering of codes and refinement of the themes.

### Public and patient involvement (PPI)

PPI work before this project involved members of VOICE, a network of public, patients and carers (https://www.voice-global.org/about/). PPI participants had expressed the importance of person-centred care, and that spiritual health has its place within whole person care. However, they were concerned about any extra burden on over-stretched primary care staff, and subsequent issues regarding access to urgent healthcare. Participants felt that doctors should not be expected to provide spiritual health support, although they noted its importance to health. Therefore, research was undertaken into social prescribing as a potential way to meet spiritual health needs. A further meeting with VOICE to discuss this specific part of the project and how best to publicly disseminate findings is planned for September 2025.

### Ethics

Ethical approval was obtained from Newcastle University on 21st August 2024 and HRA approval was obtained on 25th October 2024, IRAS number 347636. Ethical consent was obtained from participants for participation, analysis and publication. All ethical practice was undertaken in accordance with the Declaration of Helsinki.

## Results

Due to the small sample size, participant demographics are not reported as this may compromise participant anonymity. However, summary details of participant roles and settings are in Table 2. The majority of participants were white and female. There are no data regarding the social prescribing workforce demographics, but anecdotally this would reflect the workforce population. Of those who gave a belief, five were spiritual but not religious, and three were Christian.

**Table 2:** Summary of participant details

ID	Role title (self-defined)*	Approximate years in role	Setting
Participant 1	Social prescriber	3.5	Semi-rural
Participant 2	Enhanced social prescriber/wellbeing coach	1	Not specified
Participant 3	Social prescriber	1.5	Not specified
Participant 4	Social prescriber - team lead	4.5	Urban
Participant 5	Social prescriber	3	Rural
Participant 6	Social prescriber	3	Suburban
Participant 7	Manager of specialist social prescribing service	4	Semi-rural
Participant 8	Social prescriber	2	Suburban
Participant 9	Social prescriber/care coordinator	1.5	Rural
Participant 10	Social prescriber	8	Urban
Participant 11	Community link worker	2	Rural
Participant 12	Social prescriber	6	Urban

*roles in social prescribing have many titles, and participants gave their role title

### Inclusion of spiritual health discussions within current social prescribing practice

Participants described feeling comfortable with the topic of spiritual health, and its place in wider healthcare. All participants appeared to find the term spiritual health meaningful and could discuss how and where it fits into their practice as social prescribers, coming under aspects of meaning, purpose and wider wellbeing.

*“Actually*,* faith for some people*,* it actually makes them who they are. It’s almost a part of their own being. So it’s – I think it’s really important that we never miss that side of people and there’s a sense of erm*,* connection. It’s very different in terms of spiritual health [mmm] that sometimes get missed and I think*,* for some people*,* that’s what lights their world. That’s what makes them who they are.” (Participant 4)*.

### Use of community faith-based organisations

Participants disclosed how community faith-based and spiritual organisations (CFBOs) are often part of their social prescribing offer. However, this was usually as part of a wider range of activities offered by these organisations to promote social wellbeing or support statutory services (e.g. lunch clubs, venues for vaccinations), rather than for spiritual care.

*“I might end up sending them to a craft group or something at a church…men can go along and get a free sandwich if they want*,* there’s food parcels there*,* they’ve got clothes. And you know*,* and I’ve had people saying before*,* “it’s a church*,* but I’m not religious.” And I said*,* “but it doesn’t matter. Anybody’s welcome to go.” (Participant 9)*.

*“We’ve established links with some of our local mosques and we’ve been running vaccination clinics in the mosques. They really*,* really got on board and it seems like the relationship is really positive and two-way which is helpful”. (Participant 10)*

Study participants mentioned the relationships they had developed with secular organisations in their local areas. However, when it came to faith-based or religious organisations the social prescribers noted their own lack of knowledge, although some mentioned that they ‘should’ or perhaps were open to make these connections:

*“It’s something that the team we’ve said about*,* that maybe reaching out to them [faith communities] and sort of building those connections a little bit… that’s something that I think maybe in the next couple of years we’re looking to sort of develop locally.”* (Participant 6).

Study participants mentioned that they would like information about religions and local faith groups to be provided to them, which contrasted with the active role that participants took in finding out about secular organisations that had no connection to religion or faith.

Participants described accompanying patients to CFBOs or visiting beforehand. Many considered this to be their sufficient usual approach to community organisations, alongside the lawful safeguarding requirements on organisations. Few sought any additional reassurances, with the responsibility for ‘due diligence’ being on patients. However, faith-based groups which focussed on community benefit, rather than proselytisation were preferred.

*“ I attend lots of churches and groups and*,* yeah*,* I’ve not witnessed where*,* you know*,* somebody is becoming forceful about talking about religion or Christianity or Islam. I’ve never seen it. It’s all about well-being of individuals. The focus is… about helping each other and supporting each other…” (Participant 12)*.

Participants in rural or suburban areas also mentioned practical issues, such as limited numbers of non-Christian faith organisations locally, or wheelchair access, that hindered their referral options:

*“Because of where we live[…] the religious support that’s in the area is more[…] typical kind of Christian… in terms of sort of culturally*,* it’s not diverse at all. And I feel like there isn’t, that there isn’t really anywhere for those [non-Christian] communities. So*,* that’s a barrier.”* (Participant 5).

However, Christian settings may also feel more familiar, and this could be linked to the cultural background of the social prescriber, as well as their majority population served.

*“Because obviously if you’re referring to a church for example*,* you’re gonna be fairly confident that that’s*,* you know*,* a safe environment for someone to go to.” (Participant 3)*.

### Barriers and facilitators to inclusion of spiritual health

#### Religion is a sensitive topic

All participants discussed patients’ spiritual health primarily through a religious lens. However, when discussing their own spiritual health, they viewed it as wider than religion, for example being a “tree hugger” (Participant 11). A few mentioned the maxim of not discussing religion or politics, and therefore it was perceived as a sensitive topic requiring professional caution:

*“I don’t speak about it too much because it could be quite a sensitive topic or it feels quite personal*,* and you wouldn’t want to accidentally offend or assume or anything*,* similar to sort of politics… perhaps it’s not the best thing to talk about.”* (Participant 8).

Those who did differentiate between spiritual health overall and religion appeared more comfortable with the topic, seeing it as a universal need rather than part of the taboo “not politics or religion”:

*“So*,* if you say faith and spirituality… it’s not that deep. It really ain’t. It’s about how you have a conversation… the broadest conversation of what is spiritual health… You can broaden it*,* you can keep it really big so it opens up the conversation and dialogues to realise that faith and spirituality probably – everyone’s got a level of it.” (Participant 4)*.

However, for other participants, there appeared to be trepidation regarding deeper or more existential conversations. Participants appeared guarded and keen to convey that they were professional and kept boundaries with their patients. The existential topics of life for some appeared to be ‘off limits’, and there was a sense of steering away from ‘deep’ discussions.

*“I haven’t ever*,* honestly*,* been able to have this kind of a conversation with anyone before*,* so I’m struggling to find the right language.” Participant 10*.

*“And I think particularly*,* I have to feel*,* I feel like I have to be would have to be careful about that around*,* you know*,* religion and churches*,* because I don’t think anybody ever wants to think they were being pushed into something.” (Participant 5)*.

Participants prioritised a patient led ‘what matters to you’ approach in their conversations, and that they don’t ‘take a history’ as such. They were clear that they prefer to await a patient cue before discussing spiritual health. Unlike other topics routinely covered, such as finances etc., some felt that it was up to the patient themselves to find help for spiritual needs, which one participant highlighted could lead to neglect of spiritual needs.

*“Well because spiritual it’s somebody’s choice*,* if they want it they’ll go out and find it*,* but it shouldn’t be suggested to you I don’t think.” (Participant 1)*.

Participants may feel more comfortable exploring spiritual health as part of wider mental wellbeing, with an overlap between mental health and spiritual health, especially around concerns about a sense of purpose and social connections, and particularly relevant for those who are bereaved, lonely or unemployed.

*“We tend to talk to people about their mental health*,* which in a way is spiritual health… if you frame it in such a way that it’s about your mental health and about your*,* it’s about helping you by being aware of your surroundings and taking that benefit from being outside*,* that’s generally well received.”* (Participant 11).

Participants raised concerns around the potential negative impact on patients who may have had previous poor experiences with religious groups, for example the trauma of rejection. While as mentioned above, many participants weren’t concerned about organisations proselytising at lunch or other clubs, they may be concerned if they were advising patients to use spiritual organisations to address spiritual needs.

*“Personally*,* I wouldn’t be actively signposting people you know who are quite vulnerable. Obviously*,* I know some a lot of groups just mean well*,* but I think if they’re going through difficult times in their life*,* the last thing they need is somebody you know*,* essentially taking them under their wing… to have that religion pushed on them.” (Participant 9)*.

Connections with local faith organisations and the delivery of inclusive community activities helped to reduce these stereotypes around religion and religious groups.

#### Is it my role as a social prescriber?

While some participants viewed spiritual health as outside of their role as social prescribers, perhaps due to its sensitivity, others viewed spiritual health as an integral part of their holistic offer. The latter perspective appeared to be more likely if their working model, often referred to as a ‘wheel of wellbeing’ included spiritual health as a ‘spoke’. A ‘wheel of wellbeing’ is a structure used in social prescribing to pictorially demonstrate different areas of wellbeing. These ‘wheels’ appear to vary geographically [27–29].

*“I would probably never bring up spiritual health… We tend to focus on mental health… what affects anxiety and depression and social isolation.” (Participant 11)*.

Even when the importance of spiritual health was acknowledged by the worker, one participant felt spiritual health discussions were prohibited.

*“I think there’s a policy that we can’t talk about it… but I wouldn’t say that with confidence*.” (Participant 2).

Some participants felt they lacked training or knowledge in the area of religion and spirituality, especially with those with beliefs different to themselves.

*“I am aware that I don’t have the sort of multicultural knowledge that I perhaps should… it would be good to connect with different faith groups and know a bit more about*,* you know*,* the groups and activities that they offer.”* (Participant 1).

#### Social prescribers’ own beliefs

The social prescriber’s own views and opinions regarding the topic of spiritual health was a key facilitator, or barrier, in discussions around spiritual health.

Participants’ own beliefs facilitated discussions around spiritual health and affected which ‘types’ of spiritual practice they were comfortable discussing, for example some felt more comfortable when their beliefs may be concordant with that of patients. However, there appeared to be greater comfort with discussing yoga, mindfulness, and meditation (spiritual practices that are less traditional in the UK) than prayer or church attendance, even when those practices were not concordant with their own.

*“I’m sort of open to conversations about [spiritual health] … I feel connected to that side of things*,* that sort of spirituality…I guess I’m very open to it.”* (Participant 8).

*“I think the – me*,* I find it erm*,* more – not easy but easy to talk about practical signposting into faith communities [yeah] and the spiritual health side of things is more – pro-*,* probably more of the – I don’t want to say New Age spirituality but more [no] conversations of spirituality [yeah] which is really interesting as I say that because I’m*,* I’m not part of the New Age spirituality.” (Participant 2)*.

Participants who were confident in distinguishing the topic of spirituality from that of religion were more likely to raise the subject of spiritual health with patients and to see its value:

*“I think if you mentioned spiritual health*,* a lot of people initially would think of religion or church or something like that. But it’s around*,* sort of*,* just the wellness of someone’s spirit*,* isn’t it really? And that can be sort of*,* maybe if they’re practicing mindfulness*,* for example… something like that*,* that can enhance your spiritual feeling*,* can’t it? So*,* I do talk to clients about that sometimes.” (Participant 6)*.

The topic of spiritual health was again portrayed as sensitive, personal, and one that should be brought up by the patient rather than the participant. It was also a source of discomfort for the social prescribers when patients directly asked about their own beliefs. Within the interviews all but one of the social prescribers shared their own beliefs – whether religious, spiritual, or none – without being prompted or asked about this, despite often acknowledging early in the interview a stigma against sharing their beliefs.

*“I used to be Christian and sort of going to the church and attending that quite a bit*,* and that was more because I was growing up. […] More lately*,* I haven’t*,* and I question my faith a bit more. I’m not sure where you would fit me if you were to choose a faith. I’m just open*,* I guess—just open to learning now… Even the staff you work with*,* sometimes you don’t know what their faith is unless you ask them.” (Participant 8)*.

## Discussion

### Summary of findings

This study aimed to explore the current practice of those who work in social prescribing regarding discussion of spiritual health, and inclusion of spiritual health within the social prescribing intervention. We also sought to identify perceived barriers and facilitators to the discussion and inclusion of spiritual health within social prescribing. Social prescribers interviewed were generally comfortable with the topic of spiritual health and found it meaningful. Signposting patients to local services is central to the social prescribing role [30] and CFBOs were included in participants’ social prescribing offers. Use of CFBOs was discussed in terms of their community-based work – for example the provision of support groups or coffee mornings – as discrete from religion. There were gaps in the connections between the social prescribers interviewed and faith-based organisations, however participants mentioned a desire to establish or build upon these connections in the future. Across our interviews, faith-based organisations were generally framed in Christian terms, although this may have reflected the predominant cultures in which participants worked.

Barriers and facilitators to discussion included the topic of spiritual health being viewed as sensitive and outwith the social prescribing role, and social prescribers’ own views on religion and/or spirituality. Participants whose own perception of spiritual health embraced concepts such as overall wellbeing and a sense of purpose considered the topic of spiritual health to align with their social prescribing role. Concordance was noted amongst participants who were involved in activities such as yoga or mindfulness, and who also appeared comfortable with topic of spiritual health. This has been noted in earlier studies which identified fewer barriers to discussing spiritual health between patients and physicians when there was alignment with religious/spiritual beliefs [31, 32]. The topic of religion appeared to feel ‘off limits’ for other social prescribers, and when faith groups were mentioned – such as community groups runs by local churches – some participants were keen to stress the secular nature of these groups. Social prescribers place importance on the interpersonal relationship and the patient-centred approach [33]. However, where current study participants appeared comfortable to lead discussions in order to evoke ‘what matters’ to their patients, patients were – conversely – expected to broach the subject of spiritual health themselves. This has previously been noted with comments by primary care practitioners [34, 35]. However, this approach puts the onus on patients to raise the topic of spiritual health, and could lead to inequitable access to holistic healthcare [35, 36]. 

Despite the stigma around discussing spiritual beliefs noted by most participants, all bar one of the social prescribers interviewed mentioned their own personal beliefs within their interviews. This appears to be an example of ‘pluralistic ignorance’ [37] where participants believed that while *they* may be comfortable with spiritual health, society at large is not. Our data hinted at underlying concerns that religion and spirituality are often conflated, with religion seen as a taboo topic within a UK context. This could lead to discomfort with the topic of spiritual health, despite underlying openness and understanding of the topic and local provision from social prescribers. Whether this strengthened the belief that some practices (for example mindfulness) are more acceptable and less stigmatised because they are divorced from their faith origins, is unclear [38]. Rather than approaching spiritual health competencies as a personal learning point, it may be that a change in systemic culture towards the topic would allow practitioners to feel able to use competencies they may already possess through their patient centred approach.

### Comparison with other literature

This is a novel study looking at attitudes towards spiritual health within social prescribing. This builds on a previous survey which identified very similar barriers and facilitators to the discussion of spiritual health [39]. This study adds depth to our previous survey data, exploring current practice, and stigma around the topic of spiritual health, as well as understanding the bias towards non-traditional or non-western spiritual practices [39]. We found similar concerns around causing offence to patients. However the interviews also suggested that there may be a fear of deep or existential conversations [39]. The legitimacy of spiritual-but-not-religious practices within healthcare (such as mindfulness and yoga) has been noted elsewhere [40–42]. The recent Theos report into the inclusion of faith-based groups into social prescribing reported similar findings [41], but from the perspective of faith groups rather than social prescribing. The Theos Think Tank found a ‘fear of religiosity’; that faith groups can be trusted as community anchors, providing referrable activities such as food banks, community groups, exercise classes, choirs, lunch clubs, and pastoral care [41]. However, despite the resources that CFBOs bring such as buildings and established networks, they face barriers to integration within social prescribing such as language and administrative challenges, which our participants did not identify. The Theos report considers faith-based groups exclusively, whereas our participants were asked to discuss spiritual health more widely, so they discussed access to nature and other provision alongside CFBOs [41]. 

In contrast to studies with GPs who voiced concerns about safeguarding issues in CFBOs [5, 35], and vulnerability of patients to cults and exploitation, our participants felt that organisational safeguarding coupled with the legal system was sufficient. This may be because doctors have a duty of care to ensure that referrals are made to suitable settings [43], whereas social prescribers viewed that it was the patient and organisation’s job to ensure due diligence was done.

### Strengths and limitations

This study provides a deeper understanding of how spiritual health fits within social prescribing in the UK. The semi-structured interview method enabled space for participants to discuss their own stigma towards the topic and elicit the pluralistic ignorance noted, while maintaining focus on the research questions. While the study included participants from a wide geographic area, including Scotland and England, participants were largely similar in terms of ethnicity and background. Due to the lack of data on social prescribers working in the UK, it is not known whether this is representative of those working in social prescribing. We did not recruit any social prescribers from Wales or Northern Ireland, which limits the conclusions that can be drawn for the devolved nations. There was selection bias towards those with an interest in the topic of spiritual health, however this was deliberate to enable a depth of data to be gathered. Participants were purposefully recruited from diverging spiritual and religious backgrounds, to add depth to the data. This is a novel piece of research that explores how the somewhat taboo topic of spiritual health is addressed with patients within social prescribing, which is itself a relatively new role within primary healthcare settings. The mix of urban and rural settings in which participants delivered their social prescribing intervention allowed for consideration of the influence of common local faith communities, and access to spiritual health provision.

### Implications for research and practice

This study provides insights into the barriers and facilitators for the inclusion of spiritual health within social prescribing. The need for further training on the topic of spiritual health to overcome barriers and stigma has been identified previously [39]. This study adds to our understanding of the pluralistic ignorance surrounding the topic of spiritual health, and the need for a systemic cultural approach to training.

The bias towards non-traditional, non-western spiritual practices poses a challenge (noted within the Theos report), that needs greater exploration. While the UK is growing more secular [44, 45], the aging population still claims a religious identity [45, 46]. In the 2021 census, 77% of those over 65 identified as having a religion in England and Wales [46]. Practitioner bias towards avoiding spiritual health could result in inequity of access to appropriate spiritual health provision for those who are religious.

## Conclusion

Social prescribing has previously been suggested as a way for patients to access support for spiritual health needs that have been identified by GPs [35]. Within the current study, barriers and facilitators included the influence of social prescribers’ own beliefs, and the beliefs that they attribute to others working in primary care. The expectation that patients will raise the topic if spiritual health is relevant, may lead to inequity of spiritual health provision. Patients may also perceive some stigma around discussing spiritual health, and prefer practitioners (such as doctors, nurses etc.) to raise the topic [47–49]. If unchallenged, this discourse and practice may result in spiritual health discussions, which are now more widely understood to be an important part of overall health, being missed within social prescribing interventions.

## Supplementary Information

Supplementary Material 1.

Supplementary Material 2.

Supplementary Material 3.

## Data Availability

Data are saved on Newcastle University secure servers and may be available in negotiation with the first author. While participants were not consented to allow public sharing of this data, data are available upon reasonable request to the authors.

## Abbreviations

GP: General Practitioner
NHS: National Health Service
PPI: Public and Patient Involvement
CFBO: Community Faith Based Organisation
