# Supplementary material for: ‘Obviously, you can’t outright ask’: what are the barriers and facilitators to discussion of spiritual health within social prescribing? A study using semi-structured interviews
Source: BMC Prim Care. 2025 Dec 10;27:10. doi: 10.1186/s12875-025-03060-0 (PMC12801478; doi:10.1186/s12875-025-03060-0)
Supplement: Supplementary file 1 — Supplementary Material 1. [file 12875_2025_3060_MOESM1_ESM.docx]

**This protocol has regard for the HRA guidance and order of contents**

**FULL/LONG TITLE OF THE STUDY**

What is the current practice, barriers and/or facilitators for the inclusion of spiritual health in social prescribing- semi-structured interviews. Part of the SHARP- Spiritual Health Assessment and Referral in Primary Care project.

**SHORT STUDY TITLE / ACRONYM**

Understanding social prescribing and spiritual health via semi structured interviews

**PROTOCOL VERSION NUMBER AND DATE**

Version 4 21^st^ October 2024

**RESEARCH REFERENCE NUMBERS**

| **IRAS Number:** 347636 |  |
| --- | --- |
| **SPONSORS Number: 202425 5 Whitehead** |  |
|  |  |

# SIGNATURE PAGE

The undersigned confirm that the following protocol has been agreed and accepted and that the Chief Investigator agrees to conduct the study in compliance with the approved protocol and will adhere to the principles outlined in the Declaration of Helsinki, the Sponsor’s SOPs, and other regulatory requirement.

I agree to ensure that the confidential information contained in this document will not be used for any other purpose other than the evaluation or conduct of the investigation without the prior written consent of the Sponsor

I also confirm that I will make the findings of the study publicly available through publication or other dissemination tools without any unnecessary delay and that an honest accurate and transparent account of the study will be given; and that any discrepancies from the study as planned in this protocol will be explained.

| **For and on behalf of the Study Sponsor:** | | |
| --- | --- | --- |
| Signature:  ...................................................................................................... |  | Date: ....../....../...... |
| Name (please print):  ...................................................................................................... |  |  |
| Position: ...................................................................................................... |  |  |
| **Chief Investigator:** | | |
| Signature: ...................................................................................................... |  | Date: ....../....../...... |
| Name: (please print):  ...................................................................................................... |  |  |

#

# LIST of CONTENTS

| **GENERAL INFORMATION** | **Page No.** |
| --- | --- |
| HRA PROTOCOL COMPLIANCE DECLARATION | i |
| TITLE PAGE | ii |
| RESEARCH REFERENCE NUMBERS | ii |
| SIGNATURE PAGE | iii |
| LIST OF CONTENTS | iv |
| KEY STUDY CONTACTS | v |
| STUDY SUMMARY | v |
| FUNDING | vi |
| ROLE OF SPONSOR AND FUNDER | vi |
| ROLES & RESPONSIBILITIES OF STUDY STEERING GROUPS AND INDIVIDUALS | vi |
| STUDY FLOW CHART | vii |
| SECTION | |
| 1. BACKGROUND | 1 |
| 2. RATIONALE |  |
| 3. THEORETICAL FRAMEWORK |  |
| 4. RESEARCH QUESTION/AIM(S) |  |
| 5. STUDY DESIGN/METHODS |  |
| 6. STUDY SETTING |  |
| 7. SAMPLE AND RECRUITMENT |  |
| 8. ETHICAL AND REGULATORY COMPLIANCE |  |
| 9. DISSEMINATION POLICY |  |
| 10. REFERENCES |  |
| 11. APPENDICES |  |

# KEY STUDY CONTACTS

| Chief Investigator | Orla Whitehead, Newcastle University, Newcastle upon Tyne NE1 7RU orla.whitehead@newcastle.ac.uk |
| --- | --- |
| Study Co-ordinator | As above |
| Sponsor | Newcastle University Newcastle upon Tyne NE1 7RU |
| Funder(s) | The John Templeton Foundation  300 Conshohocken State Road, Suite 500  West Conshohocken, PA 19428 USA  Phone: +1610-941-2828 Fax: +1610-825-1730 |
| Key Protocol Contributors | Professor Barbara Hanratty, Population Health Sciences Institute Newcastle University [barbara.hanratty@newacstle.ac.uk](mailto:barbara.hanratty@newacstle.ac.uk)  Professor Amy O’Donnell, Population Health Sciences Institute Newcastle University [amy.odonnell@newcastle.ac.uk](mailto:amy.odonnell@newcastle.ac.uk) |
| Committees | SHARP project advisory group can be contacted via Dr Stacey Rand: s.e.rand@kent.ac.uk |

**STUDY SUMMARY**

| Study Title | What is the current practice, barriers and/or facilitators for the inclusion of spiritual health in social prescribing- semi-structured interviews. Part of the SHARP- Spiritual Health Assessment and Referral in Primary Care project. |
| --- | --- |
| Internal ref. no. (or short title) | Understanding social prescribing and spiritual health via semi structured interviews |
| Study Design | Semi-structured qualitative interviews |
| Study Participants | Volunteer participants from staff who work within ‘social prescribing’- this may involve a variety of job titles, such a ‘social prescriber’, ‘link worker’, ‘care coordinator’ or more local titles. |
| Planned Size of Sample (if applicable) | Between ten and twenty interviews. |
| Planned Study Period | 1^st^ November 2024 to 28^th^ February 2025 (Interviews will be completed by 1^st^ February 2025)  The Sharp Project as a whole is funded until 31^st^ December 2025, and therefore this study will have had all analysis and dissemination completed by then. |
| Research Question/Aim(s) | What is the current practice of social prescribing workers regarding identifying patient’s spiritual health needs?  What are the barriers and facilitators to discussion of spiritual health with patients/service users?  What is the current practice of social prescribing workers regarding working with, or referring to, spiritual community organisations, and/or faith based organisations?  What are the barriers and/or facilitators for the inclusion of spiritual or faith based community provision within suggested ‘social prescriptions’? |

**STUDY LAY SYNOPSIS**

These semi structured interviews will explore how spiritual health is included in UK social prescribing. We will interview between 10 and 20 NHS staff who work within social prescribing. We will ask about current practice regarding spiritual health, and barriers and facilitators, both to identifying spiritual health needs, and how spiritual community organisations are included, or not, in a ‘social prescription.’

**FUNDING AND SUPPORT IN KIND**

| **FUNDER(S)**  (Names and contact details of ALL organisations providing funding and/or support in kind for this study) | **FINANCIAL AND NON FINANCIALSUPPORT GIVEN** |
| --- | --- |
| The John Templeton Foundation | Full funding for the study |
| Newcastle University | Ethical, personnel, software and IT support, sponsorship. |
| North of England Commissioning Support | Governance, support |
| Clifton Court Medical Practice | Support as NHS employer of Orla Whitehead as a general practitioner |

**ROLE OF STUDY SPONSOR AND FUNDER**

The Templeton Foundation arranged peer review of the design of this study, however they will not have any role during its execution, analyses, interpretation of the data, or decision to submit results.

The sponsor is Newcastle University, which will support the study via policies, data storage, software, insurance and indemnity. Newcastle University is the employer of the investigators and has overall responsibility for the research. The study academics are employed by Newcastle University, and their responsibilities include checking the study is well planned, and good quality, including the literature review of existing evidence already carried out. Newcastle University will provide ethical oversight, and ensure approval before the research starts. Newcastle University will ensure appropriate arrangements are made for making information about the research publicly available before it starts, will agree appropriate arrangements for making data accessible, with adequate consent and privacy safeguards, in a timely manner after it has finished; and ensuring arrangements for information about the findings of the research to be made available, including to participants, in line with the institutional arrangements.

**ROLES AND RESPONSIBILITIES OF STUDY MANAGEMENT COMMITEES/GROUPS & INDIVIDUALS**

**Study Steering Groups**

Patient & Public Involvement Group

Co-design and stakeholder involvement is core to this project, and the pragmatic approach. Stakeholders will be recruited and involved from the start, to offer expertise in their area, for example faith based expertise, or patient experience. A faith (and non-faith) based expert spiritual community advisory group will be set up to feed in to the stakeholder group, chaired by Revd Dr Stacey Rand from the University of Kent.

Academic advisory steering group consists of:

Carol Jagger, Professor emerita, Newcastle University

Alistair Appleby, Former GP, NHS Highland

Glen Milstein, City University of New York

Cindy Schmidt, Kansas City University

Gowri Anandarajah, Brown University Medical School

Wilfred McSherry, Staffordshire University

Niels Christian Hvidt, University of Southern Denmark

Salman Waqar, NHS Frimley ICB

Emily Wood, University of Sheffield

Jonathan Gallagher, Chaplain

Ross Bryson, GP

Stacey Rand (Chair)

Sarah Giffen, Chaplain

Jonathan Shapiro, former GP and academic

Esther Platt, Theos

Pali Hungin, Emeritus professor of primary care, GP

**PROTOCOL CONTRIBUTORS**

Supervisory group, advisory group

| **KEY WORDS:** | Social prescribing, primary care, spiritual health, spirituality, religion, spiritual wellbeing, saltutogensis, holistic health. |
| --- | --- |

# STUDY TIMELINE

# July 2024- finalising protocol, and obtaining required ethical and other approvals, insurance etc. Finalise interview topic guide, and pilot interviews.

# August 2024- recruitment, contact participants., and scheduling interviews

September to November 2024- conduct interviews- max 6 interviews per week, per researcher, discuss initial data at PPI group, advisory group and qualitative data group.

November and December 2024- analysis and write up findings

January 2025 onwards- submit articles to journals, and abstract to conferences.

**STUDY PROTOCOL**

What is the current practice, barriers and/or facilitators for the inclusion of spiritual health in social prescribing- semi-structured interviews. Part of the SHARP- Spiritual Health Assessment and Referral in Primary Care project.

Via 10-20 interviews, we are looking asking: what is the current practice of those working in social prescribing around spiritual health? What are the barriers and facilitators to discussion of spiritual health in social prescribing? Is the HOPE tool (a teaching structure for discussing spiritual health) likely to be acceptable and/or helpful in social prescribing in the UK?

This study will recruit from NHS staff who work in Social Prescribing, an initiative which straddles primary care and the local community to bring together community resources to help primary care patients improve their salutogenesis- complete overall health. These staff are commonly called link workers, care coordinators, social prescribers, navigators, or other terms. The interviews will be semi-structured, and ask about current practice regarding spiritual health, as well as barriers and facilitators to the inclusion of spiritual health in social prescribing. This will be analysed, and then disseminated and published, as well as informing the next stage of the SHARP project.

# 1 BACKGROUND

## Context and History

The GMC (General Medical Council- the regulator of UK doctors) expect doctors to consider all the religious, social and cultural factors that are either relevant to a patient’s problem,[1,2] or the way in which the problem is addressed.[2] The Royal College of General Practitioners (RCGP) includes spiritual health in their curriculum for GP trainees,[3] specifically in sections on mental illness and palliative care.[3] Discussing spiritual health is not just required of GPs by regulators and professional bodies, it may be a beneficial intervention in itself. [4-6] It is suggested discussing spiritual health could increase job satisfaction for GPs.[7]

Religiosity and spirituality are associated with longevity[8,9], and research into the interaction between spirituality and a healthy life is a growing field.[8,10] The covid pandemic, and the resulting population distress, has arguably made spiritual health even more timely. Spirituality and/or religion has been found to be beneficial for people with mental illness,[11,12] potentially lowering stigma and loneliness for those with HIV[11] and benefiting older people with dementia.[13] Attending religious services may also enhance rehabilitation after cardiac illness.[11] While there is potential for pathological spiritual and religious beliefs and experiences, or religion led vaccine refusal, for example,[11] spirituality can be a resource for health and wellbeing.[14,15] Unmet spiritual needs can be detrimental to health,[16] including greater pain,[17,18] increased mortality,[19] a desire for hastened death[20] and lower wellbeing.[21] If good spiritual health does lead to a longer and/or healthier life, then holistic health care, including spiritual health, should be extended beyond patients who are mentally ill or approaching the end of life.

Social prescribing has been developed in UK primary care for patients who need ‘social’ interventions and it is a current major policy focus.[22] Our proposed work will complement existing initiatives to expand social prescribing after the COVID-19 pandemic.[23] In social prescribing, a local link worker provides a referral point for General Practitioners (family doctors) and other primary care professionals to redirect patients who need non-medical, more socially based interventions. Link workers are embedded in the local communities and have broad awareness of what is available from community groups- for example crafts, choirs, grief support, mental health cafés. The number of social prescribing link workers is currently expanding, as a result of state investment.[22] The aim is that social prescribing should allow the development of personalised, relational care to meet patients’ holistic needs.[24] It is also a way for GPs to access services that add meaning, purpose and connections to a patient’s life, to improve their health.[25] Meaning, purpose and connections can be considered part of spiritual health. Salutogenesis, the concept of wellbeing referred to in social prescribing literature[26], or the ‘creation of health’[27], includes the spiritual in its definition.[28] Current social prescribing initiatives are not known to include any references to spiritual care providers, or faith communities.[25,26] [29] Meeting a patient’s social, spiritual and psychological human needs should help to tackle health inequalities,[30] as community spiritual health resources typically encompass values of solidarity, justice, unity in diversity and participative decision-making.[31]

Most communities in the UK have spiritual and religious groups, but such resources may not be included in local social prescribing, despite even when there is a rich resource of expertise in spiritual support that could benefit health,[32] and other health support from faith based organisations.[33-35] Faith based organisations can find this lack of official recognition challenging,[36] although there are now an increasing number of initiatives to build relationships between faith based organisations and healthcare.[37,38] At the start of this project, we will look for evidence of current practice in initiatives in primary care to enhance spiritual health of patients. We will then go on to identify facilitators and barriers to the discussion of spiritual health for social prescribers.

Clinicians in the UK face many barriers in talking to patients about their spiritual health and in supporting patients’ spiritual health needs. In our recent work, only half of UK GPs surveyed were comfortable with the topic of spiritual health, with barriers cited of - fears of regulator or peer disapproval, lack of training, and discordance in background between practitioner and patient.[39] While there are tools (such as the HOPE tool)[39,40] that can help train GPs in spiritual history taking, the question remains, of where to send patients in need of spiritual health support. While authors have discussed the GP role as being 'priestly',[41] GPs are not trained to provide spiritual care, and patients may need guidance as to where better to access care for these needs, when GPs services are under extreme pressure. Social prescribing link workers could be the obvious link between community spiritual resources and primary health care teams. However, we do not know what current practice is for these link workers, nor what the barriers and facilitators are for the discussion of spiritual health with patients.

## How have recent endeavours of similar focus been done, and what are their limitations?

Within the UK, primary care chaplaincy services have attempted to improve patient access to spiritual health care.[42-45]The use of primary care chaplaincy may reduce demand on GP appointments,[43] and they could be viewed as an integral part of the multi-disciplinary team (MDT).[46,47] However, primary care chaplains are small in number, and this service is offered in very few areas, so access is a major limitation .

In GP training, spiritual health is included within the current UK RCGP curriculum. We contacted the RCGP about this topic, and they responded that it is a ‘good practice’ area and an ‘important aspect of care.’ Holistic care can be seen as a ‘softer, less traditional part of the training.[41] Our 2019 survey found that lack of training is a barrier to discussion of spiritual health.[39] Respondents reported that they had not had any training in this area,[39] and none of the RCGP e-learning continuing professional development (CPD) modules make any reference to ‘spiritual’. This is an important omission. A recent review of spiritual care training found one paper from the UK regarding medical student training, and one paper describing GP training from Denmark,[48] showing training is limited. Training is offered by some religious medical organisations, such as the Christian Medical Fellowship’s Saline Solution course, which is limited to their members, and has a one faith agenda. Nursing and midwifery training appears to have more content on spiritual health, for example the Enhancing Nurses' and Midwives’ Competence in Providing Spiritual Care through Innovative Education and Compassionate Care (EPICC).[49] The GMC guidance on discussing spiritual matters with patients[50] does not appear to engender confidence in the topic, as respondents named fear of regulatory involvement as a barrier to discussion, especially in cases of discordance between doctor and patient in terms of ethnicity, age or religious background.[39] This barrier to discussion is recognised in the literature.[51,52] Robust and clear training, with guidelines and a structure, e.g. the HOPE tool, is needed to help overcome such concerns.[39]

Swiss researchers argued for an embedded model of spiritual health in primary care,[53] however on informal discussions with these researchers, there are multiple barriers to implementing this at scale. There are several small pilot projects being developed in French-speaking Switzerland focused on older adults, and with people from the churches and nursing care, rather than primary care as a whole.

There are a range of tools used to assist healthcare professionals to discuss spiritual health in a structured way. The most commonly used are the FICA (**f**aith, **i**mportance, **c**ommunity and **a**ddress in care) [54] and HOPE (**h**ope, **o**rganised religion, **p**ersonal spiritual practice, **e**ffects on care).[40] A small pilot in Germany ran workshops for GPs in taking a spiritual history using a tool similar to FICA[54] as well as medical assistants in spiritual histories and directing patients.[55] Feedback was good, however this involved a five hour training session,[55] and it is doubtful such a long session would be feasible within the UK National Health Service (NHS), as GPs identified ‘time’ as a barrier to discussion of spiritual health.[39,52] While the FICA tool is referred to more frequently in the research literature, this does not appear to be due to any intrinsic strengths, it happens to be more widely known. Blaber et al’s systematic review found that the HOPE tool most comprehensively addressed the spiritual themes in a healthcare setting.[56] We have chosen to focus on the HOPE tool, as it is easily recalled as a mnemonic in the UK, it does not require a faith or religion to be relevant, and we have evidence it is acceptable to GPs.[39] It’s acceptability to social prescribing link workers will be explored in this project.

Examples have been found, of teaching in GP trainee regional teaching sessions, which take the form of brief discussions regarding barriers to discussing spirituality, and the risks and benefits of such a discussion. GMC guidance regarding ethical boundaries of discussion is given. Tools such as the FICA[54] and HOPE[40] tools may be briefly mentioned, and GP registrars have the chance to role play using these structures. A potential weakness for these sessions is that they are variable, and run by those with an interest, e.g. a strong personal faith.

Social prescribing is a relatively new development in UK primary care. While there could be areas where spiritual aspects of health are included in the local offer, data are limited. The All Party Parliamentary Group: Faith and Society published work investigating partnerships between local authorities and faith based groups in the light of the pandemic found that faith groups provided much beneficial support during the pandemic, although most of the topics discussed were more practical, e.g. food parcels, befriending.[57] There are local initiatives to bring together faith based organisation and healthcare more frequently.[37,38] They also describe similar barriers and concerns as reported from our work with GPs- concerns about equalities, and proselytising.[57]

# 2 RATIONALE

We are conducting a realist review to understand what has been tried in primary care already to improve patients’ spiritual health,and a mixed-methods online survey to obtain baseline data on spiritual health and its inclusion in social prescribing. However, to understand the context, barriers and facilitators, and what is working well, for whom and where, we need to conduct some semi-structured interviews about spiritual health with people working within social prescribing.

We will address:

The current practice of social prescribing workers in relation to

- identifying patients’ spiritual health needs, and
- working with spiritual community organisations, and/or faith based organisations

The barriers and facilitators to

- - - discussion of spiritual health with patients/service users, and
    - inclusion of spiritual or faith based community provision within suggested ‘social prescriptions’

**3** **THEORETICAL FRAMEWORK**

## This study uses semi-structured interviews, which will be analysed using thematic analysis, as Braun and Clark 2022.[58] Semi-structured interviews were chosen to allow the necessary focus on the research question in limited research time, structured around the findings from a mixed methods survey of social prescribers.[59] However, using semi-structured interviews allows participants to guide the discussion, relational data to be gathered on healthcare workers’ experience, and allows flexibility for the researcher to collect data in unforeseen areas.[59] This is particularly useful in the topic of spiritual health, and where a pragmatic approach is being taken, as spiritual health is context dependent and often very personal. There will be more than one interviewer, and there will be regular reflexive discussions around the interviews during the process, as each interviewer will bring their own biases to the data gathered. The use of more than one interviewer is planned to enrich the data.

## This interview study forms part of a wider project, SHARP, which will co-design a training intervention around spiritual health for primary care.

In the wider project, we wish to understand the attitudes towards spiritual health in primary care; and how comfortable and confident GPs and social prescribing link workers feel with discussing spiritual health . Our research so far has discovered a stigma towards the topic in UK primary care, and multiple barriers to discussing the topic with patients. This project aims to change this by further building the evidence base around the topic of spiritual health in primary care, identify barriers and facilitators to the inclusion of spiritual health in patient discussions with social prescribing link workers, and build a co-designed, pragmatic training package for the primary healthcare team on the topic.

The aims for this part of the project are to develop:

- Social prescribing link workers’ perceptions of what has worked, and what has not, when initiatives have been undertaken to enhance spiritual health in the past.
- An understanding of current practice, and the barriers and facilitators to the discussion of spiritual health for social prescribing link workers.

A pragmatic approach is taken to understanding current practice of inclusion of spiritual health within the holistic care offer in social prescribing.

# 4 RESEARCH QUESTION/AIM(S)

What is the current practice of those who work in social prescribing regarding discussion of spiritual health, and inclusion of spiritual health within the offer in social prescribing? what are the perceived barriers and facilitators to the discussion and inclusion of spiritual health within social prescribing?

**4.1** **Objectives**

- To deepen our understanding the current inclusion of spiritual health in the social prescribing offer
- To understand perceptions of barriers and facilitators to inclusion of spiritual health in the social prescribing offer
- To explore training needs of people working in social prescribing around discussion of spiritual health

**4.2 Outcome**

To provide data to inform the development of a training intervention to improve spiritual health discussion in primary care.

# 5 STUDY DESIGN and METHODS of DATA COLLECTION AND DATA ANALYIS

**Semi-Structured Interviews**- The interview guide is attached. Transcription services, Voice to text, and Nvivo software will be used for managing the data, however coloured pens, scissors and paper will also be used for analysis. It will be stored securely on Newcastle University servers. Data will be archived in line with Newcastle University policies.

# 6 STUDY SETTING

- Participants will have either a) responded to an invitation at the end of a survey or via email to contact the researcher, or b) seen a social media advert, and voluntarily got in touch to indicate their willingness to take part in an in-depth interview on the topic.
- Participants will be offered telephone or video online interview.

**7 SAMPLE AND RECRUITMENT**

**7.1 Eligibility Criteria**

Participants will work in social prescribing in UK primary care. Social prescribing is a role that straddles the wider community while being fully integrated into primary care. These staff may have titles such as ‘link worker’, ‘social prescriber’, ‘care coordinator’ or a variety of other roles. The distinctive features of these roles are that the staff see themselves as working in a ‘social prescribing’ role, work within primary care, have direct contact with patients, and signpost to community services, no matter what the particular job title.

**7.1.1 Inclusion criteria**

- Work in the UK
- Work in primary care
- See their role as within ‘social prescribing’
- Have direct patient contact

**7.1.2 Exclusion criteria**

- Those who have no direct patient contact
- Those who see their role as purely administrative (for example receptionists trained in ‘care navigation)
- No exclusion based on sex, age, ethnicity, disability, maternity, etc.

**7.2 Sampling**

Email invitations to a quantitative survey on social prescribing and spiritual health will be sent out widely throughout the UK, to care boards, practices, health boards, clinical research networks and shared via professional networks, and social media. At the end of the survey, the research team will ask for permission to contact the survey respondent again. Then, people working in social prescribing will be sent an invitation to take part in an interview. Invitations to interview will also be shared in primary care networks, via social media, professional networks etc. Throughout, an opt-in approach is used, where potential participants have to indicate a positive interest in being contacted about the research.

**7.2.1 Size of sample**

It is expected that the sample will be 10-20 interviews, however interviews will take place until data sufficiency is reached.

**7.2.2 Sampling technique**

If sufficient people come forward to be interviewed, purposive sampling will be undertaken to gain a broad range of views from social prescribers (who have volunteered to take part) . Diversity will be sought within the sample, according to working area, working life, age, ethnicity, and religious background (or none).

**7.3 Recruitment**

Participants recruited will have contacted the team to volunteer after a survey, or after seeing a social media advert, and contacted the PI to express interest in further work.

**7.3.1 Sample identification**

Participants have self identified as eligible. Eligibility will be confirmed at a screening email, where initial consent will be sought. All interviews will be conducted remotely, and participants will be given a £20 gift voucher as gratitude for their time. This voucher will be given if the participants start the interview, even if they decide to withdraw consent.

**7.3.2 Consent**

Consent will be an ongoing process- participants have implicitly consented to contact by contacting the researcher themselves, and disclosing their status as social prescribing workers. They will be invited to participate in an interview, those who express an interest will be contacted with details of consent, the nature and objectives of the study, and possible risks. They will be given the written consent information assuring anonymity. Capacity to consent will be assumed, but if the researcher has any reason to doubt capacity at the time of the interview, the interview will not go ahead. Participants will be encouraged to ask questions before the interview starts, and to stop and ask at any time during the interview.

# 8 ETHICAL AND REGULATORY CONSIDERATIONS

Ethical approval was sought and obtained from Newcastle University on 21^st^ August 2024

## **8.1 Assessment and management of risk**

Potential risks of the study:

| Potential risk | Risk management |
| --- | --- |
| Risks to the researcher if undertaking interviews alone in person, including covid risks | The immediate risk to personal safety is low as most interviews will take place remotely, online. If any researcher has any concerns for her or his safety or that of participants, the interview will be abandoned, and help sought via emergency services if needed. The supervisory team are there to support decision making, and to allow supervision of the health of the researcher. The PI is trained in use of supervision for issues of transference and counter transference and similar issues that occur when discussing difficult topics. |
| Risks to the participant | Spiritual health can be an emotive topic to discuss. Consent will be an ongoing process, and should the participant need a break, or to stop at any time, this will happen. The participant will be supported to access signposted resources and support services if need be. There may be local primary care staff support services, participants could contact their own GP practice, or support via the ‘Looking after You’ NHS service. If the researcher has concerns about safeguarding the participant if they are vulnerable, or about managing the risk, the supervisory team are there to support decision making. |
| Risks to others | It may become apparent during an interview that there is a risk to others (for example patients). All interviewers will have undertaken safeguarding level 1 training, with the PI having undertaken level 3 training. All interviewers will undertake Prevent training. If the researcher is concerned about risks to others, including risks of extremism, given the study topic, these will be discussed with the supervisory team, and decisions regarding this will be shared, with the care of the patient being our first concern. If there are concerns, the participant may be encouraged to take action themselves as a first line, before any undertaking of information sharing, however if there is immediate risk to others, or concerns that fall under Prevent, confidentiality may need to be broken in order to safeguard others. The PI is familiar with these sorts of safeguarding dilemmas, and is level 3 trained. These decisions will not be taken alone, unless an emergency, and will be shared with the supervisory team, and if appropriate, the researcher’s medical defence organisation. |

**8.2 Research Ethics Committee (REC) and other Regulatory review & reports**

The participants are social prescribers, whether currently employed or not, and so are recruited by basis of their status, and not any specific working role or employment with a particular organisation. Newcastle University ethical approval was sought and gained on the25/07/2024, and NHS REC approval is not required. Clarification is being sought as to whether HRA approval is required.

**Regulatory Review & Compliance**

- Before Newcastle University can enrol participants into the study, the Chief Investigator will ensure that all appropriate approvals are in place. Newcastle University is the only study site.
- For any amendment to the study, the Chief Investigator, in agreement with the sponsor will submit information to the appropriate body in order for them to issue approval for the amendment.

Amendments

All amendments will be handled in line with Newcastle University policies.

**8.3 Peer review**

Orla Whitehead (Chief investigator) applied for competitive funding for the SHARP project via the Templeton Foundation. This research project was presented, and received peer reviewed, and was deemed fundable.

**8.4 Patient & Public Involvement (PPI)**

The primary stakeholders in this research primary care staff, and the research is GP led with primary care participants. The study topic was discussed previously at a PPI (VOICE) group about spiritual health. They recognised the need to ‘keep spirits up’, but also that GPs were not best placed to help meet spiritual health needs. This prompted the chief investigator to develop the research project proposed. A PPI meeting with VOICE is planned for December 2024, to discuss the findings from the survey before this, and the initial qualitative data from the first few interviews. Based on this meeting, it is planned that the interview guide can be developed further, and the PPI group can inform the analysis of the interview data.

**8.5 Protocol compliance**

- Accidental protocol deviations can happen at any time. They must be adequately documented on the relevant forms and reported to the Chief Investigator and Sponsor immediately.
- Deviations from the protocol which are found to frequently recur are not acceptable, will require immediate action and could potentially be classified as a serious breach.

###

**8.6 Data protection and patient confidentiality**

Patient confidentiality will be maintained, and the study will be compliant with all GDPR regulations according to Newcastle University guidance. Data will only be collected that is proportionate, and necessary for the research. Identifiable information will be kept separately from the interview data itself, and any linking key will be destroyed at analysis. Data will be stored at data.ncl.ac.uk, however access would only be given in a limited, non-identifiable way, for secondary analysis, on discussion with the study team.

8.7 Indemnity

Newcastle University (the sponsor) will provide indemnity for the study, in line with its usual procedures.

**8.8 Access to the final study dataset**

The study team (the CI and her supervisory team) will be the only individuals with access to the full data set. Other investigators will access it to check the integrity of the research only if a formal request is received, justified and accepted by the study team. Data will be anonymised immediately, with any key kept only by the team, and destroyed at analysis stage. Secondary analysis will only be approved for limited, unidentifiable data.

### 9 DISSEMINIATION POLICY

### 9.1 Dissemination policy

- - The data will be owned by Newcastle University.
  - On completion of the study, the data will be analysed and tabulated and a Final Study Report prepared.
  - The full study report will be available from Orla Whitehead
  - Orla Whitehead and the supervisory team will publish the data
  - The Templeton Foundation will be acknowledged with their standard wording.
  - The study abstract will be shared with participants, and a copy of the final published report
  - The study protocol, full study report, anonymised participant level dataset, and statistical code for generating the results will not be made publicly available.

**9.2 Authorship eligibility guidelines and any intended use of professional writers**

Orla Whitehead intends to be the first and corresponding author, with other team members, and Barbara Hanratty as authors. Contributorship statements will be completed.

### 10 REFERENCES

# References (of literature cited in preceding sections)

1. Good Medical Practice (2013).

2. General Medical Council. Outcomes for Graduates. In: GMC, editor. London2018.

3. The RCGP Curriculum: Professional & Clinical Modules, (2016).

4. Mueller PS, Plevak DJ, Rummans TA. Religious involvement, spirituality, and medicine: implications for clinical practice [Review]. Mayo Clinic Proceedings.76(12):1225-35.

5. Yang GM, Tan YY, Cheung YB, et al. Effect of a spiritual care training program for staff on patient outcomes. Palliative and Supportive Care. 2016;15(4):434-443.

6. Borneman T, Ferrell B, Puchalski CM. Evaluation of the FICA Tool for Spiritual Assessment. Journal of Pain and Symptom Management. 2010;40(2):163-173.

7. Brémault-Phillips S, Olson J, Brett-MacLean P, et al. Integrating spirituality as a key component of patient care [Article]. Religions. 2015;6(2):476-498.

8. Zimmer Z, Jagger C, Chiu C-T, et al. Spirituality, religiosity, aging and health in global perspective: A review. SSM - Population Health. 2016 2016/12/01/;2:373-381.

9. Powell LH, Shahabi L, Thoresen CE. Religion and spirituality: Linkages to physical health. American psychologist. 2003;58(1):36.

10. Rew L, Wong YJ. A systematic review of associations among religiosity/spirituality and adolescent health attitudes and behaviors. Journal of adolescent health. 2006;38(4):433-442.

11. Litalien M, Atari DO, Obasi I. The Influence of Religiosity and Spirituality on Health in Canada: A Systematic Literature Review. Journal of Religion and Health. 2022 2022/02/01;61(1):373-414.

12. Snider A-M, McPhedran S. Religiosity, spirituality, mental health, and mental health treatment outcomes in Australia: a systematic literature review. Mental Health, Religion & Culture. 2014 2014/07/03;17(6):568-581.

13. Agli O, Bailly N, Ferrand C. Spirituality and religion in older adults with dementia: a systematic review. International Psychogeriatrics. 2015;27(5):715-725.

14. Koenig HG. Research on religion, spirituality, and mental health: A review. The Canadian Journal of Psychiatry. 2009;54(5):283-291.

15. Sohail MM, Yasin MG. A systematic review on religiosity, spirituality and health. Rawal Medical Journal. 2017;42(4):575-575.

16. Koenig H. Religion, Spirituality, and Health: The Research and Clinical Implications. Vol. 2012. 2012.

17. Harris JI, Usset T, Krause L, et al. Spiritual/Religious Distress Is Associated with Pain Catastrophizing and Interference in Veterans with Chronic Pain. Pain Medicine. 2018;19(4):757-763.

18. Grant E, Murray SA, Kendall M, et al. Spiritual issues and needs: Perspectives from patients with advanced cancer and nonmalignant disease. A qualitative study. Palliative and Supportive Care. 2004;2(4):371-378.

19. Pargament KI, Koenig HG, Tarakeshwar N, et al. Religious Struggle as a Predictor of Mortality Among Medically Ill Elderly Patients: A 2-Year Longitudinal Study. Archives of Internal Medicine. 2001;161(15):1881-1885.

20. Rodin G, Lo C, Mikulincer M, et al. Pathways to distress: The multiple determinants of depression, hopelessness, and the desire for hastened death in metastatic cancer patients. Social Science & Medicine. 2009 2009/02/01/;68(3):562-569.

21. Hills J, Paice JA, Cameron JR, et al. Spirituality and Distress in Palliative Care Consultation. Journal of Palliative Medicine. 2005 2005/08/01;8(4):782-788.

22. NHS England. Personalised care->Social prescribing [10-01-23]. Available from: <https://www.england.nhs.uk/personalisedcare/social-prescribing/>

23. National Academy for Social Prescribing. Thriving Communities [10/01/23]. Available from: <https://socialprescribingacademy.org.uk/thriving-communities/>

24. Griffith B, Pollard T, Gibson K, et al. Constituting link working through choice and care: An ethnographic account of front-line social prescribing [<https://doi.org/10.1111/1467-9566.13569>]. Sociology of Health & Illness. 2022 2022/10/25;n/a(n/a).

25. Brandling J, House W. Social prescribing in general practice: adding meaning to medicine. British Journal of General Practice. 2009;59(563):454.

26. Health Education England. Social prescribing at a glance; North West England: A scoping report of activity for the North West. 2016.

27. Rakel D. The salutogenesis-oriented session: creating space and time for healing in primary care [Review]. Explore: The Journal of Science & Healing.4(1):42-7.

28. Lindström B, Eriksson M. Contextualizing salutogenesis and Antonovsky in public health development. Health promotion international. 2006;21(3):238-244.

29. University of Westminster. Making Sense of Social Prescribing.

30. Marmot M. Social determinants of health inequalities. The lancet. 2005;365(9464):1099-1104.

31. Vader J-P. Spiritual health: the next frontier. European Journal of Public Health. 2006;16(5):457-457.

32. Tomkins A, Duff J, Fitzgibbon A, et al. Controversies in faith and health care. The Lancet. 2015 2015/10/31/;386(10005):1776-1785.

33. Johnsen S. Where's the ‘Faith’ in ‘Faith-Based’ Organisations? The Evolution and Practice of Faith-Based Homelessness Services in the UK. Journal of Social Policy. 2014;43(2):413-430.

34. Jayne M, Williams A. Faith-based alcohol treatment in England and Wales: New evidence for policy and practice. Health & Place. 2020 2020/11/01/;66:102457.

35. Livingstone P. Christians Against Poverty research into illegal lending in Northern Ireland. 2019.

36. Leavey G, Loewenthal K, King M. Challenges to sanctuary: The clergy as a resource for mental health care in the community. Social Science & Medicine. 2007 2007/08/01/;65(3):548-559.

37. Singh H. How faith groups are supporting health and social care 2023 [24/05/23]. Available from: <https://religionmediacentre.org.uk/news/how-faith-groups-are-supporting-health-and-social-care/>

38. Local Government Association. Working with faith groups to promote health and wellbeing 2017 [24/05/23]. Available from: <https://www.local.gov.uk/sites/default/files/documents/working-faith-groups-prom-6ff.pdf>

39. Whitehead IO, Jagger C, Hanratty B. Discussing spiritual health in primary care and the HOPE tool—A mixed methods survey of GP views. PLOS ONE. 2022;17(11):e0276281.

40. Anandarajah G, Hight E. Spirituality and medical practice: Using the HOPE questions as a practical tool for spiritual assessment [Review]. American Family Physician. 2001 01 Jan;63(1):81-88.

41. Pink J, Jacobson L, Pritchard M. The 21st century GP: physician and priest? The British Journal of General Practice. 2007;57(543):840-842.

42. Macdonald G. Primary care chaplaincy: a valid talking therapy? [10.3399/bjgp17X689221]. British Journal of General Practice. 2017;67(655):77.

43. Macdonald GW. Primary care chaplaincy: an intervention for complex presentation. Primary Health Care Research & Development. 2018:1-12.

44. Kevern P, Hill L. ‘Chaplains for well-being’ in primary care: analysis of the results of a retrospective study. Primary Health Care Research &amp; Development. 2014;16(1):87-99.

45. Bunniss S, Mowat H, Snowden A. Community chaplaincy listening: Practical theology in action. Scottish Journal of Healthcare Chaplaincy. 2013;16(1):42-51.

46. Professional Association of Community Healthcare Chaplaincy. The Case for Community Healthcare Chaplaincy: facilitating whole-person care in General Practice 2011.

47. Gordon T, Kelly E, Mitchell D. Spiritual Care for Healthcare Professionals. London: Radcliffe Publishing; 2011.

48. Rykkje L, Søvik MB, Ross L, et al. Educational interventions and strategies for spiritual care in nursing and healthcare students and staff: A scoping review [<https://doi.org/10.1111/jocn.16067>]. Journal of Clinical Nursing. 2022 2022/06/01;31(11-12):1440-1464.

49. EPICC Network. Enhancing Nurses' and Midwives’ Competence in Providing Spiritual Care through Innovative Education and Compassionate Care. Available from: <https://blogs.staffs.ac.uk/epicc/>

50. General Medical Council. Personal Beliefs and Medical Practice. 2013.

51. Ellis MR, Campbell JD. Concordant spiritual orientations as a factor in physician-patient spiritual discussions: a qualitative study. J Relig Health. 2005 Spring;44(1):39-53.

52. Best M, Butow P, Olver I. Doctors discussing religion and spirituality: A systematic literature review. Palliat Med. 2016 Apr;30(4):327-37.

53. Bornet M-A, Edelmann N, Rochat E, et al. Spiritual care is stagnating in general practice: the need to move towards an embedded model. British Journal of General Practice. 2019;69(678):40.

54. Puchalski CM. The FICA Spiritual History Tool #274. Journal of Palliative Medicine. 2014;17(1):105-6.

55. Kunsmann-Leutiger E, Straßner C, Schalhorn F, et al. Training General Practitioners and Medical Assistants Within the Framework of HoPES3, a Holistic Care Program for Elderly Patients to Integrate Spiritual Needs, Social Activity, and Self-Care into Disease Management in Primary Care. J Multidiscip Healthc. 2021;14:1853-1861.

56. Blaber M, Jones J, Willis D. Spiritual care: Which is the best assessment tool for palliative settings? International Journal of Palliative Nursing. 2015 09/02;21:430-438.

57. All Party Parliamentary Group: Faith and Society. Keeping the Faith 2.0. 2022.

58. Braun V, Clarke V. Thematic analysis : a practical guide. London: SAGE Publication Ltd; 2022.

59. DeJonckheere M, Vaughn LM. Semistructured interviewing in primary care research: a balance of relationship and rigour. Fam Med Community Health. 2019;7(2):e000057.

### 11. APPENDICIES

**11.1 Appendix 1- Required documentation**

CVs of the research team

PIS

Consent form

**11.2** **Appendix 2 – Schedule of Procedures**

| **Procedures** |  |  |  |
| --- | --- | --- | --- |
|  | **Screening** | **Baseline** | **Interview** |
| Informed consent | x | x | x |
| Demographics |  | x |  |
| MBI and spiritual score |  | x |  |
| Interview |  |  | x |

**13.3** **Appendix 3 – Amendment History**

| **Amendment No.** | **Protocol version no.** | **Date issued** | **Author(s) of changes** | **Details of changes made** |
| --- | --- | --- | --- | --- |
|  |  |  |  |  |
