# Supplementary material for: ‘Obviously, you can’t outright ask’: what are the barriers and facilitators to discussion of spiritual health within social prescribing? A study using semi-structured interviews
Source: BMC Prim Care. 2025 Dec 10;27:10. doi: 10.1186/s12875-025-03060-0 (PMC12801478; doi:10.1186/s12875-025-03060-0)
Supplement: Supplementary file 2 — Supplementary Material 2. [file 12875_2025_3060_MOESM2_ESM.docx]

**Identifying fraudulent research participants**

Any doubts to be discussed as a team at the earliest opportunity

namenumber@gmail addresses will be asked for further evidence of their involvement in social prescribing via email, for example the type of organisation they work for. The authenticity/inauthenticity of their response to be discussed as a team. This can be followed by further questions to ascertain their knowledge of the topics of social prescribing and spiritual health.

The interview will start with recording, understanding of the PIS, and video on. If a non-organisational email has been used, and video is off, the interview will be terminated.

The voucher will be given unconditionally for genuine participants, even if they withdraw. However it will be made clear that if we believe that they are not a genuine participant, then the interview will be terminated without remuneration. All efforts will be made through questioning to clarify this early.

Check early on that there’s a genuine understanding of primary care, the team, social prescribing, and how they fit within that.
