# Supplementary material for: ‘Obviously, you can’t outright ask’: what are the barriers and facilitators to discussion of spiritual health within social prescribing? A study using semi-structured interviews
Source: BMC Prim Care. 2025 Dec 10;27:10. doi: 10.1186/s12875-025-03060-0 (PMC12801478; doi:10.1186/s12875-025-03060-0)
Supplement: Supplementary file 3 — Supplementary Material 3. [file 12875_2025_3060_MOESM3_ESM.docx]

Understanding social prescribing and spiritual health via semi-structured interviews

IRAS number 347636

Topic Guide

Introduction:

- Purpose of interview: “This interview is entirely voluntary, and you can pause or stop at any time. My name is x, and I am x. This interview is about your role in social prescribing, and the topic of spiritual health. We plan to explore how spiritual health fits, or not, within your role as a social prescriber, and the relationship between primary care and spiritual or faith-based health services in the community. The interview will be recorded, and will take about an hour, how do you feel about that?”
- Ensure read the PIS and clarify any questions- ensure recording is understood, and that the recordings and transcripts will be anonymised and kept securely.
- Reconfirm consent, and hand over £20 gift voucher. Make sure aware can pause, or stop, any time, and keep the voucher with no further contact.
- If participant asks “what is spiritual health?” explain “Spiritual health can mean lots of things to different people. We’re interested in exploring spiritual health whatever that means to you, or your patients, in all their diversity.”

Section A (context):

- Tell me about your role in social prescribing?
  - - Length in role
    - Describe your role and the setting you work in
    - What type of people are referred to you, and who do you see and support?
    - What sort of support do you offer?
    - What type of organisations do you connect with?
    - Training undertaken

Section B (further depth, barriers and facilitators, attitude):

- What sort of discussions do you have with people around their spiritual health and wellbeing?
- What prompted these? What happened as a result? What went well? What didn’t go well?
- What is your experience of religious, spiritual or faith-based organisations in the community? Are there any religious or spiritual organisations that you link in to in your professional role? Who? How? Barriers? Etc.
  - - Safeguarding concerns
    - Cults
    - For non-spiritual activities, such as choirs?

Section C (future changes):

- If further training was developed around discussing spiritual health, what should that look like? Is there anything you feel it should or shouldn’t include
  - - Online?
    - Individual?
    - Group?
- What changes could made to improve how you respond to your patients' spiritual health needs?
  - - How could relationships be developed between community faith, religious or spiritual based organisations or providers
    - Regulatory change?
    - Culture?
